# Methods for the Improvement of Acne Scars Used in Dermatology and Cosmetology: A Review

**DOI:** 10.3390/jcm11102744

**Published:** 2022-05-12

**Authors:** Karolina Chilicka, Monika Rusztowicz, Renata Szyguła, Danuta Nowicka

**Affiliations:** 1 Department of Health Sciences, Institute of Health Sciences, University of Opole, 45-040 Opole, Poland; monika.rusztowicz@uni.opole.pl (M.R.); renata.szygula@uni.opole.pl (R.S.); 2 Department of Dermatology, Venereology and Allergology, Wrocław Medical University, 50-368 Wrocław, Poland; danuta.nowicka@umed.wroc.pl

**Keywords:** acne vulgaris, atrophic scars, dermatology, scar improvement

## Abstract

Acne vulgaris is a chronic skin disease that, depending on its course, is characterized by the occurrence of various skin eruptions such as open and closed comedones, pustules, papules, and cysts. Incorrectly selected treatment or the presence of severe acne vulgaris can lead to the formation of atrophic scars. In this review, we summarize current knowledge on acne scars and methods for their improvement. There are three types of atrophic scars: icepick, rolling, and boxcar. They are of different depths and widths and have different cross-sections. Scars can combine to form clusters. If acne scars are located on the face, they can reduce the patient’s quality of life, leading to isolation and depression. There are multiple effective modalities to treat acne scars. Ablative lasers, radiofrequency, micro-needling, and pilings with trichloroacetic acid have very good treatment results. Contemporary dermatology and cosmetology use treatments that cause minimal side effects, so the patient can return to daily functioning shortly after treatment. Proper dermatological treatment and skincare, as well as the rapid implementation of cosmetological treatments, will certainly achieve satisfactory results in reducing atrophic scars.

## 1. Introduction

Acne vulgaris is a chronic skin disease that, depending on its course, is characterized by the occurrence of skin eruptions such as open and closed comedones, pustules, papules, and cysts [1,2]. It occurs in more than 80% of adolescents, 50–60% of women aged 20–25 years, and 12% of women over 25. Factors that cause the disease include abnormal keratinization of the pilosebaceous canal, increased sebum production, bacterial colonization, as well as inflammatory and hormonal disorders of the skin [3,4].

During the course of the disease, patients with acne experience itching and burning of the skin. Acne scars appear most often in people struggling with severe forms of acne, leading to a reduction in their quality of life. Patients often feel ashamed, embarrassed, anxious, and socially isolated, which can lead to depression and even suicidal thoughts [5,6,7,8].

A scar (also named cicatrix) is a skin lesion that results from the healing of wounds due to chemical, mechanical, or thermal injuries. Scars can develop after skin inflammation, for example, as a complication of acne. Scar formation is part of the wound healing process, which is divided into three phases: inflammatory, healing, and remodeling.

The inflammatory phase begins in the first 6 h after the skin is injured and continues for the next 48–72 h. During this phase, blood vessels dilate, and their permeability increases under the influence of kinins, histamine, prostaglandins, and leukotrienes. The skin is visibly swollen and red. The exudate starts to produce itself. Leukocytes migrate to the inflamed site under the influence of chemotaxins. The wound is debrided due to the dominance of white blood cells (neutrophils) and their phagocytic properties, as well as due to the release of proteolytic enzymes. The recruitment of monocytes, which replace neutrophilic granulocytes, increases on the second day after skin injury. Subsequently, after contact with the intercellular matrix, monocytes are transformed into macrophages, due to which microbial phagocytosis and wound debridement take place. A range of cytokines and a growth factor are produced due to macrophage activation, which stimulates fibroblasts and white blood cells. All those processes stimulate epithelialization and the formation of new blood vessels [9,10,11].

The second phase of wound healing is the proliferative phase. It begins 2–3 days after tissue damage and may continue for 3–6 weeks. It lasts from about 10 days to several weeks on average. In this phase, the number of fibroblasts stimulated by cytokines (PDGF, EGT, TGF beta, FGF) increases. The process of granulation tissue formation and collagen production begins. The skin defect is filled with granulation tissue containing a network of capillaries, fibroblasts, and white blood cells. This step begins between the third and fifth day after the injury and lasts up to several weeks. First, type III collagen is produced, and then it is replaced by type I collagen. Between the 14th and 28th days after the injury, collagen synthesis is most intense, and then it decreases due to the enzyme activity of the metalloproteinases of the extracellular matrix. During the production of granulation tissue in a wounded area, epithelization occurs, and new blood vessels are formed. During epithelialization, keratinocytes enter the wound from the periphery. This is followed by the proliferation of keratinocytes [12,13].

The last phase—the remodeling phase—is the rebuilding and maturation of the scar. This begins between the third and seventh day after the injury and lasts several months up to a year. Granulation is replaced by fibrous tissue. Approximately 5 days after the injury, the wound contracts, and this process continues for approximately 14 days. Microfibroblasts, present in a wound, have contractile properties due to active filaments that create gap junctions with fibronectin and collagen. As a result of their contraction, the edges of the wound are pulled together, and as a result, the wound gets smaller [14,15].

### Types of the Acne Scars

Incorrect production and degradation of collagen during the healing process can lead to the development of various types of scars. Scars can be classified according to the cause and time of their formation and appearance. The last group of acne scars is divided into atrophic, hypertrophic, and discolored. Atrophic scars are below the surface of the skin and are recessed. On the other hand, hypertrophic scars are raised above the skin surface [16].

Atrophic scars can be divided into the following types: icepick, rolling, and boxcar. Icepick scars are deep, narrow, and can reach the border of the dermis with the subcutaneous tissue. They have sharp edges, a width of not more than 2 mm, and a V-shaped cross-section with a narrowing deep into the skin. The skin of a person with such scars looks like it has been pierced with a skewer or sharp instrument. Icepick scars account for 60% to 70% of atrophic scars. Boxcar scars are oval or round but quite wide and flat and resemble the letter U or a square. Their edges are well marked and have a demarcated edge. They are usually 0.1–0.5 mm deep and 1.5–4 mm wide. They can combine to form clusters. Boxcar scars have the cross-section of the letter M and account for 15 to 20% of atrophic scars. Rolling scars are the largest of all types and can reach a diameter of 5 mm. They comprise 15% to 25% of atrophic scars [16].

## 2. Methods of Acne Scars Management

Contemporary aesthetic medicine and cosmetology can offer a wide range of effective methods for reducing acne scars. Depending on the type of scar, its location, and the depth of the lesions, a dermatologist, in cooperation with a cosmetologist, can propose a series of treatments that will significantly flatten unwanted skin defects and improve the patient’s quality of life.

### 2.1. Ablative Laser Resurfacing

Several traditional ablative lasers are used to treat acne scars, e.g., 10,600 nm carbon dioxide (CO_2_) lasers and 2940 nm pulsed Er:YAG lasers [16]. Conventional 10,600 nm CO_2_ lasers emit light in the far-infrared spectrum. Such a laser creates many microscopic treatment zones to activate new collagen formation and re-epithelialization [17]. The first application of the continuous CO_2_ laser took place in the 1980s. Over the years, pulsed action lasers have started to be implemented, which has improved the precision of their action and depth of skin ablation and thus reduced the occurrence of side effects [18]. The CO_2_ laser, either fully ablative or fractional mode, has been proposed to treat various conditions alone or combined with other devices [19,20,21].

The study by Fang et al. suggests that in treating atrophic acne scars in Asians using a fractional CO_2_ laser, 3 treatment sessions led to better results as assessed after 3 months [22]. Several other studies indicate that combined treatments can give better results than single laser treatments alone. Li et al. also showed an improvement in skin texture in patients with atrophic scars after one treatment. In this study, fractional microplasma radiofrequency treatment combined with fractional ablative CO_2_ laser treatment was performed in 45 patients, while a single procedure was performed in 19 patients. There were no statistically significant differences between the two groups; however, a greater number of combined treatments helped improve acne scars [23]. Gala et al. conducted a study on 30 patients using a fractional CO_2_ laser on one side of the face and a fractional CO_2_ laser followed by platelet-rich plasma (PRP) intradermal injections on the other half. In both groups, the effects were satisfactory; however, the synergy of the treatments contributed to a better improvement in atrophic scars, which was shown 3 months after completion of the treatments using the skin analysis camera [24]. Zhou et al. used a combination of the CO_2_ laser with allogeneic stem cells. This treatment improved elasticity and skin hydration. A histological examination showed an increase in the density of dermal collagen and elastic fibers [25].

The traditional pulsed Er:YAG laser (2940 nm) was developed as a less aggressive alternative to the traditional CO_2_ laser. The emitted wavelength is also in the infrared range; however, the thermal energy is more limited and precise at the same time, as the laser light closely approximates the absorption peak of water (3000 nm). For this reason, all energy is absorbed in the epidermis and superficial dermis. An Er:YAG laser at 5 J/cm vaporizes tissue at a depth of 20 to 25 μm with an additional 5 to 10 μm zone of thermal necrosis. This causes less damage to the skin than when a CO_2_ laser is used [16,18,26].

Tidwell et al. compared the fractional Er:YAG laser with fully ablative Er:YAG in people with hypertrophic scars. Twenty patients with scars not younger than 8 weeks and not older than 1 year were enrolled in the study, but only 16 patients completed the entire series of treatments. A fractionated Er:YAG laser was used on one half of the face, while treatments with a fully ablative Er:YAG laser was applied on the other side. A series of 3 treatments were performed with a one-month interval between them. Follow-up visits were made 1 and 2 months after the final treatment. The study showed superior outcomes in the fractionated Er:YAG group over the fully ablative Er:YAG group [27]. Zgavec and Loca demonstrated using a histological examination that wound healing after Er:YAG treatment is shorter when compared to full beam ablative treatment with milder side effects [28]. Cenk et al. investigated the effectiveness of a series of 4 multifractional Er:YAG laser treatments in women and men with acne scars. At the end of the 4th session, the improvement rate was 26% to 50% in 14 of 24 patients and 51% to 75% in 10 patients [29]. Emam et al. treated 21 people with the fractional Er:YAG laser and radiofrequency micro-needling (MN). Er:YAG laser treatment was performed on one side of the face and radiofrequency MN on the other side. A series of 4 treatments were performed, each with a month-long break. To compare the effectiveness of the treatments, optical coherence tomography was used. After the end of the series of treatments, both sides of the face were compared, showing very good results and a scar improvement. There were no significant differences between these two devices. Optical coherence tomography showed a significant increase in epidermal and dermal thickness after 4 treatments compared to the baseline [30].

### 2.2. Non-Ablative Lasers

This group of lasers includes a pulsed dye laser (PDL) with a wavelength of 585 to 600 nm, ND:YAG with a wavelength of 1320 nm, and a diode laser with a wavelength of 1450 nm. This kind of laser is helpful in reducing scars classified as boxcar or atrophic. The chromophore in PDL is hemoglobin, and the laser supports the reduction of scars of various etiologies with the simultaneous elimination of blood vessels within the lesion. The adverse effect of using this device include persistent post-operative purpura for up to 14 days [31].

Rogachefsky et al. investigated the treatment of atrophic or a mixed pattern scarring with a 1320-nm Nd:YAG laser. Twelve patients with atrophic facial acne scars (N = 6) or a combination of atrophic and pitted, sclerotic, or boxcar scars (N = 6) underwent 3 laser sessions. The mean improvement in acne scars was 1.5 points on physician assessments (*p* = 0.002) and 2.2 points on patient assessments (*p* = 0.01). There were no complications at 6 months [32].

Sadick et al. used a 1320-nm Nd:YAG laser to check the efficacy of lasers for the treatment of acne scars. Eight probants with acne scars received 6 monthly treatments with a 1320-nm Nd:YAG laser with built-in cryogen cooling. The scar improvement was significant [33].

The 1450-nm diode laser was used by Tanzi et al. to compare it with the 1320-nm Nd: YAG laser. Twenty patients with mild-to-moderate atrophic facial acne scars randomly received 3 monthly treatments with a long-pulsed 1320-nm Nd:YAG laser on one half of the face and a long-pulsed 1450-nm diode laser on the other half of the face. The 1450-nm diode laser had a better clinical scar response [34].

Rathod et al. conducted a study on 48 probants with acne scars with a low-energy double-pass 1450-nm diode laser (5 treatment sessions). At the end of the 3rd month, 92.9% of the patients demonstrated >30% improvement. The diode laser treatment was efficient in facial acne scars when used with a double pass at low energy [35].

The studies conducted by Cannarozzo et al. deserve special attention. These two investigations showed the effect of a 675-nm laser on the skin. This device is recommended to treat hyperpigmentation, scars, and various types of wrinkles. In a case report of a 42-year-old man, after the laser treatment, skin biopsies showed the proliferation of new collagen fibers in the treated area. Histological analysis suggests that the 675-nm laser has a potential role in stimulating collagen remodeling, with a significant increase in thin and new collagen fibers. Another study by the same authors was conducted on 24 patients with acne scars. The patients had 3 sessions of the 675-nm laser. The efficacy of the treatment was evaluated using the Goodman and Baron grading scale before and 3 months after the last treatment. All patients had significant scar improvement and no side effects. This laser system is well tolerated with comfortable and easy posttreatment management for patients [36,37].

### 2.3. Trichloroacetic Acid

Trichloroacetic acid (TCA) is an organic compound of the class of carboxylic acids with the following chemical formula C(Cl)3COOH. It is the most effective and one of the strongest acids. Its power (pKa) is 0.26 and, for comparison, the pKa of glycolic acid is 3.83. Depending on the effects to achieve, superficial peeling (10–30% TCA), medium-deep peeling (35% TCA), and deep peeling (50% TCA) can be used. This acid coagulates proteins and damages living cells of the epidermis, resulting in necrosis of the epidermis and the upper layer of the dermis, as well as exfoliation of the cells. In the long term, the treatment with TCA stimulates the production of new collagen fibers. The application of TCA to the skin causes protein denaturation (keratocoagulation), resulting in the appearance of white shedding on the patient’s skin, the so-called frost [38,39].

Agarwal et al. evaluated the application of 70% TCA every 2 weeks in 53 patients with atrophic scars using the chemical reconstruction of skin scars (CROSS) technique. Good or very good improvement was seen in 66% of the patients. In total, 81.1% of the patients reported being satisfied or very satisfied [40]. Bhardwaj et al. used the same CROSS technique in 12 patients with atrophic scars but with 100% TCA every 2 weeks. Eight out of 10 patients reported an improvement in post-acne scars of more than 70%. No side effects were observed during treatment. One of the patients reported a lack of further improvement after 3 months after treatment [41]. Al-Hamamy et al. evaluated the effectiveness of 25% TCA combined with dermasanding. They reported a satisfactory improvement in acne scars. No significant complications were found except erythema and post-inflammatory hyperpigmentation, which disappeared after 3 months in affected patients [42]. Puri researched 25 patients who were divided into 2 groups. The first group had Jassner’s peeling with 20% TCA, while the latter had only 20% TCA. In the first group, mild improvement in acne scars was observed in 8% of the patients, moderate improvement was observed in 32% of the patients, and marked improvement was observed in 60% of the patients. In the group treated with 20% TCA alone, mild improvement was seen in 32% of the patients, moderate improvement was observed in 40% of the patients, and marked improvement was observed in 25% of the patients. The results in both groups were similar. The group using the combination of Jessner’s peel with 20% TCA did not show better results than the group treated with 20% TCA alone [43].

Concerning TCA, our research team is finishing researching its influence on acne scars. The results after only 2 treatments are very promising, and the skin structure is significantly improved. This study will be published soon.

### 2.4. Microneedling

MN is used in cosmetology as a treatment that rejuvenates the skin, improves its tension, and also improves stretch marks and scars. Due to the lack of post-inflammatory discoloration after MN, it is often used as an alternative to laser treatment in the case of skins with IV and V phototypes. The following MN devices can be used: rollers, stampers, and pens (electric or non-electric) [44]. Research has shown that after a series of treatments, there is increased expression of type I collagen, as well as glycosaminoglycans, vascular endothelial growth factor (VEGF), fibroblast growth factor (FGF)-7, epidermal growth factor (EGF), and transforming growth factor (TGF)-β, all important signaling molecules for collagen production and neovascularization [45,46].

In the study by El-Domyati et al., 10 patients were treated with skin MN in a series of 6 treatments performed every 2 weeks. Skin biopsies were obtained at baseline and after 3 months from the start of the treatment. The authors reported an improvement in acne scars and a significant increase in collagen types I, III, and VII [47]. Tirmizi et al. enrolled 50 patients with moderate to severe grade atrophic acne spars classified using the Goodman and Baron’s Global Acne Scarring System. Three treatments were carried out every 4 weeks. The authors concluded that after MN, scars improved together with the improvement in scar grade, resulting in a greater number of patients classified as grade II after MN compared to the baseline. The study showed a beneficial effect of MN on the patient’s skin [48]. Biesman et al. evaluated the effects of MN used in combination with Polymethylmethacrylate-Collagen Gel Dermal Filler (PMMA). In the study, 44 probants were enrolled and divided into 2 groups. The first group received 3 MN treatments and the second group received 3 MN treatments combined with PMMA. After 24 weeks, the group that had combined treatment showed better treatment effects. This fact confirmed the occurrence of synergies of treatments, so a combined treatment is advisable to achieve better results [49]. Finally, Schoenberg et al., in their study and a short review, described the effect of combined MN treatments with PRP in a group of 50 patients. The MN procedure was carried out on the entire face. Each half was treated with PRP or distilled water injected into the skin. After the end of the series of treatments, combined treatment showed better results [50].

Our experience shows that combining an MN treatment with, e.g., BioRePeel, gives much better results than performing separate treatments.

### 2.5. Fractional Radiofrequency

The use of microneedle radiofrequency is based on the ability to selectively heat the tissue at a specific depth. The device also uses micro-needles through which a beam of radio waves is emitted directly into the tissue. Additionally, the needles only heat up at their tips, which allows precise control of the depth at which the tissue is heated. Such a feature of this technique is unique and important for final effects. The puncture depth can be adjusted to 0.5 to 3.5 mm. Microneedle radiofrequency has gained great popularity in cosmetology due to improvement in scars.

Kim et al. used a fractional radiofrequency microneedle treatment in 52 patients with atrophic acne scars located on the face. Each of the patients received 4-treatment series. The Goodman and Baron’s Global Acne Scarring System evaluated treatment results. Overall, 73.1% of patients improved according to the grading system. Five patients experienced post-inflammatory hyperpigmentation. The authors concluded that this method is effective with minimal risk of complications [51]. Chandrashekar et al. used microneedle radiofrequency to treat 31 patients with moderate and severe acne scars. The study protocol was based on 4 treatments conducted over 6 months every 6 weeks. After the treatments, the scars improved. Of grade 3 and 4 acne scars, 80.64% improved by 2 grades, and 19.35% improved by 1 grade, according to the Goodman and Baron’s Global Acne Scarring System. The transient adverse effects reported by the patients were pain, erythema, edema, and hyperpigmentation [52]. Elawar et al. recruited 19 patients to evaluate the improvement in acne scars and skin, as well as the reduction of skin pores after 2 to 4 microneedle fractional radiofrequency treatments conducted at intervals of 1 month. The authors concluded that this method effectively treats acne scars, as it improves skin texture, reduces pore size, and increases patient satisfaction. Furthermore, none of the patients experienced hyper/hypopigmentation [53]. Kim et al. aimed to investigate the effectiveness of bipolar fractional radiofrequency in patients with acne scars and enlarged pores. The study protocol was based on 4 treatments conducted at 3-week intervals. The evaluation was carried out 3 months after completion of the protocol and showed clinical improvement. Objective measures confirmed improved elasticity and the melanin/erythema index, as well as more procollagen types I and III, and elastin. Furthermore, bipolar fractional radiofrequency appeared to be safe, as none of the patients reported side effects [54]. Similar observations were made by Qin et al. after a series of 4 treatments performed every month on a group of 23 Asian patients. The acne score improved significantly at 4 and 12 weeks after the first treatment. Side effects included transient pain, erythema, and dryness of the skin [55]. Chilicka et al. in their case reports showed that it is extremely important to continue research on the effects of glycolic acid and fractional mesotherapy to reduce acne scars. The needle radiofrequency with the application of active substances provided very good results for the shallowing of post-acne scars. This therapy changed the structure, visual appearance of the skin, and made the scars more shallow, as analyzed using the Goodman and Baron’s scale. In addition, only needle radiofrequency gave good results in shallowing the scars [56,57]. It should also be remembered that to prevent acne scars it is extremely important to care for the skin of people who suffer from acne. This is evidence-based on numerous scientific studies [58,59,60,61]. Combination treatments are very useful in scars and improve scars much better than a single treatment (Table 1).

### 2.6. Platelet-Rich Plasma

Platelet-rich plasma (PRP) is an autologous blood-derived product enriched in platelets, growth factors, and chemo/cytokines delivered in a volume of plasma. PRP has the potential to deliver a high concentration of growth factors to the target tissues [69]. Lee et al. conducted a split-face study with 14 probants from Korea. They underwent fractional laser resurfacing, and the researchers randomly assigned them to receive normal saline or PRP. The improvement after 4 months was superior in the group with laser and PRP [62].

Mumtaz et al. compared the efficacy of PRP versus 50% TCA using the CROSS technique in treatment; PRP was significantly better than 50% TCA in reducing post-acne atrophic scars [64]. Bhargava et al. conducted a study using PRP as adjunctive therapy to a combined subcision and needling treatment in severe atrophic acne scarring. Thirty patients were randomly divided into 2 groups (15 patients for each group). Group A underwent 3 treatments-subcision and needling. Group B had 3 subcision, needling, and topical application treatments of PRP. Scar improvement ≥50% was better in group B than group A patients (*p* = 0.025) [70].

Ibrahim et al. used skin MN plus PRP versus skin MN alone to treat 35 patients with mild-to-severe acne scars. The patients received 4 sequential treatments of skin MN alone on the right side of the face and skin MN followed by topical application of PRP on the left side of the face. Treatment was conducted at intervals of 3 weeks. A significant improvement was observed on both sides of the face [67].

Nilforoushzadeh et al. used autologous fat, stromal vascular fraction cells, and PRP as cell therapy techniques in atrophic acne scars. Nine patients received autologous fat transplantation, PRP, and stromal vascular fraction cells. After 6 months, there was an improvement in skin lightness, skin elasticity, transepidermal water loss, spots, and skin pores. More than 66% of the patients were satisfied after these treatments [68].

Chawla S. used MN with PRP on one side of the face and MN with vitamin C on the other side. The treatments had an interval of 1 month. Overall, 27 out of 30 patients completed the study. The PRP group achieved a better response than the MN with the vitamin C group. The patients treated with MN and vitamin C were also more satisfied [71].

### 2.7. Subcision and Punch Techniques

#### 2.7.1. Subcision

Subcision is based on inserting the needle under the scar to sever the fibrous tissue and binding down the scar. This method releases fibrous tissue and raises the scar so that it is effective in rolling and other tethered scars. Ebrahim et al. conducted a study to evaluate the efficacy of subcision versus its combination with cross-linked hyaluronic acid or poly-l-lactic acid threads in atrophic post-acne scars. Forty men and women took part in this study based on 3 sessions conducted at 1-month intervals. Subcision combined with hyaluronic acid or threads gave better clinical improvement than subcision alone [63].

Nilforoushzadeh et al. conducted a pilot study with 9 patients with acne scars. The probants underwent subcision with the Endolift (200-nm fiber). The study showed a 90% improvement. The number of lesions before the treatment was 25.5 ± 12.1, and after treatment, it was reduced to 11.4 ± 2.1 (*p* < 0.05) [65].

Deshmukh et al. used PRP and the subcision method in their study. Forty patients completed the split-face study. The right side of the face was treated with autologous PRP injected after subcision. The left side was the control side, where only subcision was performed. The right side, after treatment, showed better improvement in post-acne scars than subcision alone. This treatment performed better for rolling scars than for box scars. It shows that synergic treatments are better for treating scars [66].

#### 2.7.2. Punch Elevation and Punch Excision

Punch elevation is the method developed for broad (3 mm) boxcar scars with sharp edges and normal bases. It is a technique where the scar is punched down to the subcutaneous tissue without being discarded. “The punched scar is then elevated and sutured in place at a level slightly higher than the surrounding skin to account for contraction during wound healing” [72].

Punch excision is suited for icepick and narrow (B 3 mm) boxcar scars. “The scar is excised down to the subcutaneous fat with the help of a punch instrument that is slightly larger than the scar, and the defect is closed with sutures along relaxed skin tension lines” [73].

## 3. Comparison of Methods

Notable is the comparison of various methods to reduce acne scars. Husein-El Ahmed et al. included 5 studies in their meta-analysis on non-ablative erbium versus CO_2_ lasers for atrophic acne scars. The scar improvement was similar for both types of laser in terms of investigator-reported scar improvement. The ablative laser produced a slightly greater response compared to non-ablative lasers based on the physician’s assessment [74].

Elsaie et al. conducted a study with 58 probants comparing these two methods. Each group included 29 patients, and each had 4 treatment sessions at 3-week intervals. The follow-up visit was after 3 months. Group A was treated with CO_2_ lasers, and group B with a 1540-nm erbium glass laser. The improvement was higher in group A, but with a non-significant difference between both treatments. These two lasers were good for treating acne scars [75].

Reinholz et al. conducted a study with 14 patients with severe scars. Both cheeks were treated randomly 4 times with lasers. One side with Er:YAG, and the other side with CO_2_ laser. The high-resolution, 3D small-field capture system, digital photography, and POSAS were used before and after the treatment sessions. The higher efficacy (better skin smoothing) was achieved in the group treated with the fractional CO_2_ laser [76].

There is a lot of research on the use of TCA in the literature. Comparing the studies by Agarwal et al. in which they used 70% TCA CROSS, and the studies by Puri et al. with modified Jessner’s Peel and 20% TCA versus 20% TCA peel alone, we can conclude that better results were obtained in the case of 70% TCA CROS [40,43].

Ahmed et al. used the CO₂ laser pinpoint irradiation technique versus CROSS technique for treating ice pick acne scars. Twenty-eight probants with acne scars were divided into 2 groups. Fourteen patients received pinpoint irradiation by CO₂ laser, and another 14 probants received TCA CROSS. Each patient had 4 sessions with 3-week intervals (3 months of follow-up). The researchers showed that clinical improvement was better with CO₂ laser [77].

Leheta et al. investigated the efficacy of combining a 1540-nm non-ablative fractional laser with percutaneous collagen induction (PCI) and 20% TCA) in treating atrophic acne scars. The results showed that the 1540-nm non-ablative fractional laser in alternation with PCI and 20%TCA effectively treats atrophic acne scars [78].

The study carried out by Nofal et al. included 45 patients with acne scars. Group A had intradermal injections of PRP, group B had a CROSS technique with 100% TCA, and group C had a combination of skin needling and PRP. Each group underwent 3 sessions (2-week intervals). The results showed that group C had the highest significant improvement [79].

El-Domyati et al. showed that combined treatment of a dermaroller and PRP or dermaroller and 15% TCA resulted in a better improvement when compared to a dermaroller. In addition, in the combined groups, the collagen bundles were better organized [80]. This study shows that combined treatments are much better than treatments conducted alone.

Fractional radiofrequency has also been used in scar reduction treatments. Currently, researchers are trying to combine treatments to increase their effectiveness. Rongsaard et al. used a 1550-nm fractional erbium-doped glass and a fractional bipolar radiofrequency to treat atrophic acne scars. Twenty patients participated in this study, and had 3 split-face monthly treatments. Fractional bipolar radiofrequency on one side of the face and 1550-nm fractional erbium-doped glass on the other side of the face was applied. This study concluded that fractional bipolar radiofrequency and fractional erbium-doped glass have similar effectiveness for scars. The pain was higher with fractional bipolar radiofrequency, while the duration of scab shedding was longer in erbium-doped glass [81].

Chae et al. conducted a study on the efficacy and safety of the 1550-nm Er:Glass fractional laser and fractional radiofrequency microneedle device. Each group (20 patients) received 3 treatments (4-week intervals) using the Er:Glass fractional laser or fractional radiofrequency microneedle device. Scars improved in both groups, but the laser was a more effective treatment than the radiofrequency microneedle [82].

Lan et al. in 2018 used micro-plasma radiofrequency to treat facial acne scars. Ninety-five patients participated in this study, and had 3 sessions of treatment at 2-month intervals. Eighty-six probants finished all sessions, 15 of 86 patients showed more than 75% improvement, 57 patients showed 50–75% improvement, and 14 patients showed 25–50%. The study confirmed that this treatment is safe and effective for acne scars [83].

Lan et al. in 2021 compared fractional micro-plasma radiofrequency and fractional microneedle radiofrequency for the treatment of scars in 60 patients. One treatment was conducted on the left part of the face, and another treatment, on the right part. The total sessions consisted of 3 treatments at 2-months intervals. Fractional micro-plasma radiofrequency was more effective for atrophic scars than fractional microneedle radiofrequency [84]. Zhang et al. compared fractional microplasma radiofrequency technology and CO_2_ fractional laser in 33 Asian patients. This was a randomized split-face treatment. The patients received 3 sessions of treatment. There was no significant difference between these methods, but postinflammatory hyperpigmentation was observed with the CO_2_ fractional laser. The microplasma radiofrequency might be better in patients with darker skin [85].

## 4. Conclusions

Acne scars are still an unpleasant complication for people who have suffered from acne vulgaris. They represent a real challenge for dermatologists and cosmetologists. Scientific research on improving acne scars is ongoing and shows multiple effective modalities to treat this type of scar. Ablative lasers and other modern devices that use, for example, radiofrequency or MN, as well as TCA, bring very good treatment results. However, it is worth noting that each scar reduction method has advantages and disadvantages. Nevertheless, research shows that the best results can be obtained through the synergy of treatments, for example: combining a 1540-nm non-ablative fractional laser with PCI and 20% TCA or combined treatment of dermaroller and PRP or dermaroller and 15% TCA. It should be remembered that each therapy should be selected individually for each patient. The best results in scar reduction are achieved with treatments using lasers, radiofrequency, MN, and PRP [24,32,47,55,78,80].

Contemporary dermatology and cosmetology use treatments that cause minimal side effects, so the patient can return to daily functioning shortly after treatment. Proper dermatological treatment and skincare, as well as the rapid implementation of cosmetological treatments, will certainly allow satisfactory results in reducing atrophic scars.

## Figures and Tables

**Table 1:** Combination treatments for acne scars.

Type of Treatment				
**Combination treatments**	Platelet-rich plasma	CO_2_ laser	Acids	Subcision
	Fractional laser resurfacing [62]	Fractional microplasma radiofrequency [23]	Dermasanding [42]	Cross-linked hyaluronic acid or poly-l-lactic acid threads [63]
	Chemical reconstruction of skin scars technique [64]	Platelet-rich plasma [24]	Jessner’s peeling [43]	Endolift (200-nm fiber) [65]
	Subcision and needling [62]	Allogeneic steam cell [25]	Fractional radiofrequency [57]	Platelet-rich plasma [66]
	Microneedling [67]			
	Transplantation of autologous fat, stromal vascular fraction cell [68]			

## Data Availability

No new data were created or analyzed in this study. Data sharing is not applicable to this article.
